# Determinants of anemia among children under five in Eastern Uganda: a community-based cross-sectional study

**DOI:** 10.3389/fpubh.2025.1618395

**Published:** 2025-08-13

**Authors:** Joel J. Komakech, Edirisa J. Nsubuga, Jessica M. Graves, Oladayo E. Apalowo, Rahel Mathews, Leah B. Pylate

**Affiliations:** ^1^Department of Biochemistry, Nutrition and Health Promotion, Mississippi State University, Starkville, MS, United States; ^2^Department of Animal and Dairy Science, Mississippi State University, Starkville, MS, United States

**Keywords:** anemia, children 6–59 months, associated factors, Modified Poisson Regression, cross-sectional study, Busoga Region, Uganda

## Abstract

**Background:**

Anemia is a leading contributor to child morbidity and mortality in Sub-Saharan Africa. In Uganda, more than half of the children under 5 years are affected by anemia. Understanding context-specific determinants remains critical for targeted interventions. This study examined the community prevalence and factors associated with anemia among children aged 6–59 months in the Busoga Region of Uganda.

**Methods:**

This community-based cross-sectional study included 439 caretaker-child dyads with children aged 6–59 months. Multistage random sampling was used to select households, and one eligible child was recruited. A pre-tested electronic face-to-face interviewer-administered questionnaire was used to collect the data. Capillary blood samples from each child were obtained through a finger or heel prick, and hemoglobin concentration was measured using standardized HemoCues. The United Nations Children's Fund (UNICEF) cut-offs were used to determine anemia status among children after adjusting for altitude. Bivariate and multivariable Modified Poisson Regression tested the association between key demographic variables and child anemia using unadjusted and adjusted prevalence ratios (APRs). Variables with *p* ≤ 0.2 at bivariate analysis were included in the multivariate regression models, using a backward stepwise method. Covariates with *p* < 0.05 were considered risk factors for anemia.

**Results:**

The mean age of the caretakers was 31 ± 11 years, with only 19 (4.3%) males. More than half (52.0%) of the children were male. Among the sampled children, 229 (52.2%) had a history of malaria, 212 (48.3%) were not dewormed, 296 (67.4%) were anemic, 22 (5.0%) had severe anemia, and 161 (36.7%) had moderate anemia. Children had a higher risk of anemia if they had a history of malaria (APR = 1.2 [1.1–1.4], *p* = 0.023), and had a caretaker aged between 45 and 59 years (APR = 1.5 [1.1–2.1], *p* = 0.025). Conversely, children aged 36-47 and 48-59 months (APR = 0.6 [0.5–0.8], *p* = < 0.001, and APR = 0.6 [0.5–0.8], *p* = 0.006, respectively) were less likely to be anemic.

**Conclusions:**

Anemia among children is a severe public health problem in the Busoga Region. It was associated with having a history of malaria, child age, and caretaker age. Interventions such as the distribution of long-lasting insecticidal nets, malaria vaccination, chemoprophylaxis, and sensitization of caregivers on adequate child feeding practices could reduce the anemia burden in the region.

## Background

Globally, the World Health Organization (WHO) estimates that anemia affects 40% of all children aged 6–59 months (269 million), and it's responsible for ~50 million child deaths (1). Anemia is a condition in which the number of red blood cells (RBCs) or the hemoglobin (Hb) concentration within the RBCs is lower than normal (1). Furthermore, iron deficiency anemia (IDA) is one form of anemia characterized by low iron levels, accompanied by microcytic hypochromic erythrocytosis, which remains a widespread global public health issue. The WHO classifies anemia prevalence rates of ≤ 4% as generally acceptable, 5.0%−19.9% as mild, 20.0%−39.9% as moderate, and ≥40% as a severe public health problem (2). Specifically, the United Nations Children's Fund (UNICEF) and the WHO describe that a child aged 6–59 months is anemic if their blood hemoglobin concentration is < 11.0 grams/deciliter (g/dl) (1–3), mildly anemic if their Hb concentration is between 10.0 and 10.9 g/dl, moderately anemic with the Hb concentration of 7.0–9.9 g/dl, and severely anemic as having a Hb concentration < 7.0 g/dl (2, 3).

However, the WHO recommends adjusting hemoglobin concentrations for altitude and smoking before applying the cut-offs, as failure to do so may lead to an underestimation of the prevalence of anemia (4, 5). When measuring a person's hemoglobin (Hb) level at high altitudes, the recommended correction ranges require adjusting by subtracting 0.2 g/dl from the Hb reading for people living at 1,000 m to 4.5 g/dl for those living at 4,500 m or higher due to the lower oxygen levels at high altitudes (2). These guidelines increase the validity of anemia assessments to inform public health interventions with higher prevalence rates, requiring a multisectoral ecological approach integrating nutritional, medical, and policy-driven strategies (6–8).

Anemia is most prevalent in low- and middle-income countries (LMICs), especially with an increased illness burden among preschool-aged children and young women (1, 5). Some of the different types of anemia include vitamin B12 deficiency, iron deficiency, aplastic anemia, folic acid deficiency, sickle cell anemia, and hemolytic anemias, among others (5, 9–11). An extensive review across 187 nations between 1990 and 2010 reported that IDA remained the primary etiology of anemia (2). Africa, with an estimated 103 million children aged 6–59 months affected by anemia, is the region most affected worldwide (1). Furthermore, Sub-Saharan Africa has an estimated prevalence of 64.1% of anemia among children aged 6–59 months (11, 12). Although prevention measures have reduced global rates of IDA, its incidence is currently highest in Central and West Africa, as well as South Asia (2, 3).

Recent WHO estimates indicated that 51.7% of children aged 6–59 months in Uganda suffer from anemia (13). The Ministry of Health (MoH) in Uganda's 2023 report highlighted anemia among the top five causes of under-five child morbidity and mortality (14). Yet the causes of anemia in children are multifactorial, encompassing various physiological and pathological mechanisms. Nutritional deficiencies, including iron, vitamin B12, and folic acid, constitute primary contributors (6). Hemoglobinopathies, such as sickle cell disease, represent significant inherited causes, while malabsorption syndromes impair nutrient absorption and utilization. Physiological factors include increased requirements during rapid growth phases and blood loss from various sources; for example, infections such as malaria, helminths, tuberculosis (TB), and human immunodeficiency virus (HIV) significantly contribute to anemia through various mechanisms (10, 15). Further, chronic diseases such as leukemia suppress normal hematopoiesis, leading to anemia (1, 6). Additional risk factors of anemia in children reported from previous studies include but are not limited to poor socioeconomic status, household food insecurity, crowded household conditions, household size, maternal education, maternal health status, household head, residence, suboptimal infant and young child feeding practices, child sex, child age, and child's birth order among others (7, 9, 10, 12, 16). Understanding these diverse causal factors is essential for implementing targeted therapeutic and preventative interventions.

Childhood anemia has both severe short- and long-term consequences on child growth, including mental, physical, social development and survival. The proximal implications of anemia in children include the impairment of a child's immune system, increased poor motor and cognitive development, and the risk of mortality (10). The distal repercussions include absenteeism in school related to morbidity, poor academic performance, and reduced productivity in adulthood, which decreases their earning potential and increases their susceptibility to poverty, causing a vicious circle (1, 16). In 2023, the Busoga Region in Eastern Uganda was identified as one of the six major hotspots of anemia (17), indicating a potentially high ongoing prevalence of anemia in the region. Therefore, this study examined the prevalence of anemia and identified the factors associated with anemia among children aged 6–59 months in the Busoga Region of Uganda, aiming to inform the design of region-specific interventions to address the anemia issue, which may be applicable to similar locales.

## Methods

### Study design, area, and period

In this study, we conducted a community-based cross-sectional study using quantitative data collection approaches in the Busoga Region of Uganda between August and September 2024. The Busoga Region is situated in eastern Uganda, ~60 miles east of Kampala, Uganda's capital. The region is bordered by Lake Kyoga in the north, Lake Victoria in the south, Victoria Nile River on the west, and Mpologoma River on the east (18). It is composed of 12 districts, including Bugiri, Bugweri, Buyende, Iganga, Jinja, Jinja City, Kaliro, Kamuli, Luuka, Mayuge, Namayingo, and Namutumba districts (18, 19). Multistage random sampling was employed to select areas within the districts of Iganga and Buyende in the Busoga region for the study to be conducted. Iganga district is ~1,120 m above sea level (20), while Buyende District is ~1,080 m (20). With 4,363,295 (9.8%) of the total population of Uganda, the Busoga Region is the second most populated region in Uganda after the Buganda Region (19). Similar to the other areas in Uganda, the Busoga Region has a higher population of females than males, with a population of 2,306,501 (52.9%) females (19). Additionally, an estimated 551,821 children aged 0–5 years make up 12.6% of the entire population of the Busoga Region (19).

### Study population

The study population comprised of children aged 6–59 months residing in households within the Busoga Region of Uganda. The primary respondents were the child caretakers paired with the selected child to be included in the study.

### Sample size and sampling procedure

An adjusted sample size of 439 caretaker-child dyads was calculated using the formula for the calculation of sample size for cross-sectional studies by Kish Leslie (21) while considering the expected prevalence of 52% of anemia in Uganda (13), a 5% level of significance, a design effect of 1.0, and a potential non-response rate of 12.5%. Multistage random sampling was used to select two out of 12 districts (Iganga and Buyende districts) in the Busoga Region, two sub-counties from each district, two parishes from each sub-county, and four villages from each parish. Probability proportionate to size sampling was used to determine the number of eligible households to be selected from each village, and random sampling was used to select eligible households and one eligible child from households with more than one child aged 6–59 months.

### Inclusion and exclusion criteria

All households in Iganga and Buyende districts with at least one child aged 6–59 months were eligible for inclusion in the study. Additionally, only one child was selected from households with more children within the 6–59 months age category. Eligible children whose caretakers were unavailable after two follow-up visits for consent for participation and those who reported being ill were excluded from the study.

### Study variables

#### Dependent variable

Anemia was defined as having a hemoglobin concentration of < 11.0 g/dl in children aged 6–59 months. Children with a hemoglobin concentration of 10.0–10.9 g/dl were mildly anemic, 7.0–9.9 g/dl were identified as moderately anemic, and those with a hemoglobin concentration < 7.0 g/dl were classified as severely anemic (2, 3).

#### Independent variables

These were identified through an extensive literature review and included sociodemographics, household food availability, child demographics, feeding practices, and health characteristics (15, 17, 22–25). The sociodemographic factors assessed were the caretaker's sex, age, marital status, and highest level of education, maternal parity, household size, sub-county of residence, category of household head, and the main source of income of the household head. Household food availability factors included the household dietary diversity score (HDDS), household food consumption score (FCS), household food insecurity access scale score (HFIAS), and household water insecurity experiences (HWISE). The child demographics and feeding practices included child sex, age, breastfeeding/breast milk consumption history, individual dietary diversity score (child IDDS), and feeding frequency. The health-related characteristics included age-specific vaccination status, history of diarrhea infection and malaria diagnosis within the 2 weeks preceding the study; vitamin A supplementation, deworming, and history of measles infection within the 6 months of the study's commencement; and micronutrient supplementation.

### Data collection, quality assurance, and quality control

Six trained research assistants and one supervisor, fluent in English and Lusoga (the local language), with medical clinical backgrounds at the graduate or postgraduate level, having experience in research methodologies, phlebotomy, and electronic data collection, were recruited to collect the data.

A pre-tested, validated, semi-structured, interviewer-administered electronic questionnaire using KoboCollect software was used to collect the data. The electronic questionnaire included inbuilt mandatory fields, validations, and skip logic, ensuring high-quality data collection. It was written in English and translated into Lusoga, the local language widely used in the study area. Each research assistant also had a printed copy of the translated questionnaire to assist in the interview process.

### Blood sample collection and assessment

Capillary blood samples were collected from each child through a finger or heel prick using sterile, single-use disposable lancets. The blood samples were drawn into microcuvettes and placed onto strips. These strips were subsequently inserted into portable digital hemoglobinometers (HemoCue Hb 301 System), which provided the hemoglobin concentration in grams per deciliter. The hemoglobin concentration reading was adjusted for altitude. The adjusted value was used to classify the child's anemia status.

### Data analysis

Data cleaning and analysis were conducted using STATA version 14 software. Descriptive statistics were used to summarize data on anemia and the independent variables. The prevalence of anemia was calculated by dividing the number of anemic children by the total number of children in the study and expressing the result as a percentage. Modified Poisson Regression analysis was used at bivariate analysis to generate unadjusted prevalence ratios (UPRs) and at multivariate analysis to generate adjusted prevalence ratios (APRs). Prevalence ratios were used instead of odds ratios at multivariate analysis because the prevalence of anemia was more than 10% and using odds ratios in such scenarios tends to overestimate the strength of the association (26, 27). The associations between anemia and the independent variables were determined using the unadjusted and adjusted prevalence ratios (UPRs and APRs, respectively), along with their 95% confidence intervals. The variables with *p* ≤ 0.2 at bivariate analysis were included in the final model, which used a backward stepwise method. The covariates with *p* < 0.05 were considered risk factors for anemia.

### Ethical approval

This study was approved by the Institutional Review Boards of Makerere University School of Public Health Research and Ethics Committee [SPHREC] (SPH-2024-599) and Mississippi State University (IRB-24-217). Additional permission was acquired from the District Health Offices (DHO) and the Chief Administrative Offices of the Iganga and Buyende districts in Uganda. Informed consent was obtained from all respondents at recruitment for participation in the study. The participants were provided with a 1 kg bar of washing soap, 200 mL of vitamin A fortified cooking oil, and half a kilo each of iodized salt and sugar, all worth 8,000 Uganda shillings (1.5 USD) as compensation for participation in the study.

## Results

### Sociodemographic and household food availability characteristics of the respondents

A total of 439 caretaker-child dyads successfully participated in the study, resulting in a 97.5 % response rate. Among the participants, 420 (96.7%) of the caretakers were females, 371 (84.5%) of the caretakers were biological mothers of the children, 202 (46.0%) of the mothers were grand multiparous with a history of four or more pregnancies, and 376 (85.6%) of the child caretakers were married/cohabiting (Table 1).

**Table 1:** Sociodemographic and household food availability characteristics of respondents in a study assessing the predictors of anemia among children aged 6–59 months in Busoga Region, Uganda.

**Characteristic**	**Frequency (*n* = 439)**	**Percent (%)**
**Sub-county**		
Bugaya	96	21.9
Bulamagi	124	28.2
Buyende town council	119	27.1
Nakalama	100	22.8
**Caretaker sex**		
Female	420	96.7
Male	19	4.3
**Caretaker age (years) (min** = **15, max** = **80) mean** ±**SD (31** ±**11)**		
15–25 (Young adult)	156	35.5
26–44 (Adult)	236	53.8
45–59 (Middle–age)	33	7.5
≥60 (Old age)	14	3.2
**Caretaker type**		
Biological mothers	371	84.5
Father/elder brother	12	2.7
Grandmother	49	11.2
Auntie	7	1.6
**Maternal parity**		
Prima parity	89	20.3
Low multiparity (2–3)	148	33.7
Grand multiparity (≥4)	202	46.0
**Marital status of child caretaker**		
Married/cohabiting	376	85.6
Divorced/separated	42	9.6
Single	9	2.1
Widowed	12	2.7
**Household size**		
2–3	77	17.5
4–5	154	35.1
≥6	208	47.4
**Highest level of education of child caretaker**		
Primary	244	55.6
Secondary	142	32.3
No formal education	35	8.0
Professional certificate (certificate, diploma, degree, etc.)	18	4.1
**Household head**		
Father	336	76.5
Mother	18	4.1
Other relatives	85	19.4
**Main source of income of the household head**		
Formal employment	32	7.3
Laborer (wage earner)	106	24.1
Agricultural/fishing	201	45.8
Trade	72	16.4
Transport business	28	6.4
**Household dietary diversity score (min** = **1, max** = **12), mean ± SD (6 ± 2)**		
Good (≥6)	225	51.0
Poor (< 6)	214	49.0
**Food consumption score (min** = **1, max** = **103), mean ± SD (52 ± 18)**		
Acceptable (≥35)	364	82.9
Borderline (21.5–34.9)	58	13.2
Poor (< 21.5)	17	3.9
**HFIAS (min** = **0, max** = **23) mean** ±**SD (6** ±**6)**		
0–9 (Food secure)	311	70.8
10–18 (Marginally food insecure)	110	25.1
19–27 (Food insecure)	18	4.1
**HWISE (min** = **0, max** = **23) mean** ±**SD (2** ±**4)**		
0–12 (Water secure)	419	95.4
13–24 (Marginally water insecure)	20	4.6

max, maximum; min, minimum; SD, Standard Deviation; HFIAS, household food insecurity access scale score; HWISE, household water insecurity experience.

The mean household size was 6 ± 3 members, with 208 (47.4%) households having six or more members, 244 (55.6%) of the caretakers having attained primary education as the highest level of education, 336 (76.5%) households being headed by child fathers, and 201 (45.8%) of the household heads having agriculture and or fishing as their primary source of income (Table 1). Out of the 439 households, 225 (51.0%) had a good HDDS, 364 (82.9%) had an acceptable FCS, 311 (70.8%) were food secure, and 419 (95.4%) were water secure (Table 1).

### Child demographics, feeding practices, and health-related characteristics

Of the 439 children recruited into the study, 230 (52.4%) were males, 128 (29.2%) were 12–23 months old, and the mean age of all the children was 27 ± 14 months (Table 2).

**Table 2:** Child demographic, feeding practices, and health characteristics in a study assessing the predictors of anemia among children aged 6–59 months in Busoga Region, Uganda.

**Characteristic**	**Frequency (n)**	**Percent (%)**
**Child sex**		
Female	209	47.6
Male	230	52.4
**Child age (min** = **6, max** = **59) mean** ±**SD (27** ±**14)**		
6–11	74	16.9
12–23	128	29.2
24–35	111	25.3
36–47	81	18.4
48–59	45	10.2
**Child breastfeeding/breast milk consumption history**		
Yes	427	97.3
No	12	3.0
**Child IDDS** ^+^		
Good (≥6)	267	60.8
Poor (< 6)	172	39.2
**Child feeding frequency**		
≥4	140	31.9
3	196	44.6
1–2	103	23.5
**Child fully vaccinated**^‡^ **at his/her age**		
Yes	415	94.5
No	24	5.5
**Diarrhea in the last 2 weeks**		
No	276	62.9
Yes	163	37.1
**Malaria in the last 2 weeks**		
No	210	47.8
Yes	229	52.2
**Measles in the last 6 months**		
No	263	59.9
Yes	176	40.1
**Vitamin A in the last 6 months**		
Yes	222	50.6
No	217	49.4
**Deworming in the last 6 months**		
Yes	227	51.7
No	212	48.3
**Current micronutrient supplement use**		
Yes	89	20.3
No	350	79.7
**Current iron supplement use**		
Yes	3	0.7
No	436	99.3

+ IDDS child infant dietary diversity score,

‡ Child meeting all current required vaccinations for their age.

Nearly all children, 427 (97.3%), had been breastfed or received breastmilk, 267 (60.8%) achieved a good individual dietary diversity score (child IDDS), and 140 (31.9%) were fed four or more times daily (Table 2).

Most of the children, 415 (94.5%) were fully vaccinated for their age, 163 (37.1%) had a history of diarrhea in the 2-weeks preceding the study, 210 (47.8%) had a history of malaria diagnosis in the 2-weeks preceding the study, 176 (40.1%) had a history of measles infection in the 6-months preceding the study. Approximately half of the children, 222 (50.6%), had received vitamin A supplementation in the 6 months prior to the study. In the same time frame, 212 children (48.3%) did not receive deworming tablets, and 89 children (20.3%) were on some form of micronutrient supplementation, with 3 (0.7%) specifically receiving iron supplementation (Table 2).

### Prevalence of anemia among children aged 6–59 months in Busoga Region

The prevalence of anemia among the children was 67.4% (95% CI: 62.8%−71.8%), with 113 (38.2%; 95% CI: 32.6%−44.0%) having mild anemia, 161 (54.4%; 95% CI: 48.5%−60.2%) having moderate anemia, and 22 (7.4%; 95% CI: 4.7%−11.0%) having severe anemia (Table 3).

**Table 3:** Prevalence of anemia among children aged 6–59 months in Busoga Region, Uganda.

**Classification of anemia**	**Frequency (*n*)**	**Percent (95% CI)**
Normal (Hb ≥ 11.0g/dl)	143	32.6 (28.2–37.2)
Anemic (Hb < 11.0g/dl)	296	67.4 (62.8–71.8)
Mild anemia^*^ (Hb = 10.0–10.9 g/dl)	113	38.2 (32.6–44.0)
Moderate anemia^*^ (Hb = 7.0–9.9 g/dl)	161	54.4 (48.5–60.2)
Severe anemia^*^ (Hb = < 7.0 g/dl)	22	7.4 (4.7–11.0)

* Among 296 children with anemia.

### Factors associated with anemia among children aged 6–59 months

Multivariate analysis showed that, the risk of anemia among children who had a history of a malaria diagnosis in the 2-weeks preceding the study was 20 percent higher than that among children who had no history of malaria in the 2-weeks preceding the study (APR: 1.2; 95% CI: 1.1–1.4, *p* = 0.023) (Table 4). Similarly, the risk of anemia among children who had caretakers aged 45–59 years was 50 percent higher than that among children whose caretakers were aged 15–25 years (APR:1.5; 95% CI: 1.1–2.1, *p* = 0.025) (Table 4). Conversely, the risk of anemia among children aged 36–47 months and 48–59 months was 40 percent lower for both age groups compared to that among children aged 6–11 months (APR: 0.6; 95% CI: 0.5–0.8, *p* = < 0.001, and APR: 0.6; 95% CI: 0.5–0.8, *p* = 0.006, respectively) (Table 4).

**Table 4:** Predictors of anemia among children aged 6–59 months in Busoga Region, Uganda.

**Characteristic**	**Unadjusted PR (95% CI)**	***p*-value**	**Adjusted PR (95% CI)**	***p*-value**
**Caretaker age (years)**				
15–25 (Young Adult)	1.00		1.00	
26–44 (Adult)	0.9 (0.8–0.9)	0.041^*^	1.1 (0.9–1.3)	0.369
45–59 (Middle-aged)	1.0 (0.8–1.3)	0.973	1.5 (1.1–2.1)	0.025^*^
≥60 (Old age)	1.1 (0.8–1.5)	0.583	1.7 (1.0–2.9)	0.061
**Malaria**				
No	1.00		1.00	
Yes	1.2 (1.1–1.4)	0.012^*^	1.2 (1.1–1.4)	0.023^*^
**Child age**				
6–11	1.00		1.00	
12–23	1.0 (0.8–1.1)	0.670	1.0 (0.8–1.1)	0.677
24–35	0.9 (0.8–1.1)	0.324	0.9 (0.7–1.1)	0.186
36–47	0.6 (0.5–0.8)	< 0.001^***^	0.6 (0.5–0.8)	< 0.001^***^
48–59	0.6 (0.5–0.9)	0.004^**^	0.6 (0.5–0.8)	0.006^**^

* *p* < 0.05,

** *p* < 0.01,

*** *p* < 0.001.

Adjusted PR (Adjusted prevalence ratio): Model adjusted for sub-county of residence, maternal parity, marital status of caretaker, type of caretaker, household size, child breastfeeding/breast milk consumption history, child feeding frequency, child age-specific vaccination status, diarrhea history, measles history, household food insecurity access scale score (HFIAS), household water insecurity experience (HWISE), and micronutrient supplementation.

## Discussion

This study examined the prevalence of anemia and risk factors among children aged 6–59 months in the Busoga Region of Uganda. The prevalence of anemia among children aged 6–59 months in the current study (67.4%) was higher than the WHO estimated national prevalence of 51.7% (13). These findings were consistent with the WHO classification of anemia as a severe public health problem in the Busoga Region and across Uganda (28, 29). The higher prevalence of anemia in the Busoga Region compared to the national prevalence may be due to higher economic deprivation, coupled with the extensive large-scale cultivation of sugarcane at the expense of food crops, contributing to household food insecurity (30). Further, the income generated from the sale of sugarcane may be insufficient to meet all household needs (17, 31). The prevalence of anemia in the current study aligns with a study conducted in Uganda, which found that the Busoga Region was one of the primary anemia hotspots in the country and had the highest risk of anemia in the eastern part of Uganda (17). The prevalence of anemia in this study is similar to findings from studies conducted in Tanzania that showed 67%−69% of children aged 6–59 months in Temeke District, Dar es Salaam (32, 33). On the contrary, the prevalence of anemia in this study was higher than in previous studies conducted in Ethiopia (46.4%−57.6%) (34–36), in Somalia 43.4% (37), in Zimbabwe 29.6% (38), and 49.5% in Mozambique (39). The difference could be due to several factors, including, but not limited to, variations in study design, sampling procedures, geographic location, and socioeconomic factors.

In this study, a history of a malaria diagnosis in the 2 weeks preceding the study increased the risk of anemia among children aged 6–59 months. These findings could have been due to the destruction of erythrocytes and the reduction of their production in the bone marrow caused by the plasmodium parasites (40). Similar findings were also reported in several other studies carried out in Togo (41), Malawi (42), Mozambique (43), and Ghana (44), which also reported that malaria infection was a statistically significant risk factor for anemia among children aged 6–59 months.

The findings in this study showed that having a caretaker aged 45 to 59 years increased the risk of anemia among children aged 6–59 months. Caretakers in this age range may have numerous additional responsibilities beyond caring for the child, including, but not limited to, supervising other children in the home and elsewhere while engaging in income-generating activities and fulfilling other duties. These findings align with a study conducted in the USA, which indicated that factors related to child caretakers, such as authoritarian and indulgent feeding styles, were associated with poor nutritional outcomes for children (45). A child caretaker is a person who provides daily care for a child, attending to basic needs such as bathing, feeding, grooming, and dressing, while also serving as the child's guardian in the absence of their biological parents (46). Moreover, they oversee the child's education, healthcare, and extracurricular activities. Child caretakers are often the child's biological parents, older siblings, grandparents, guardians, or other relatives. However, non-relatives may also be employed to care for the child in their absence (46). These individuals are commonly referred to as nannies in the developed world (45).

In this study, children aged 36–47 months and 48–59 months had a lower risk of being anemic compared to those aged 6–11 months. This could be due to the better-developed digestive systems of children aged 36–59 months, who are fully weaned and thus have access to a broader variety of iron-rich family foods whenever available (47). In contrast, infants aged 6–11 months depend on their mothers or caregivers to provide iron-rich foods to meet their increased iron needs, as breast milk alone is insufficient to fulfill their recommended dietary allowances (RDA) of ~11 mg per day, starting at 6 months (48). Our findings align with a study conducted in Botswana, which revealed that older age groups of children under 5 years have a lower risk of anemia than their younger counterparts (49). Moreover, our findings are consistent with results from previous studies conducted in Uganda (50), Ethiopia (51), Cape Verde (52), and Sierra Leone (53), which reported a higher risk of anemia among children aged 6–24 months compared to older children aged 36–59 months.

### Study limitations

This study had some limitations, including potential recall and social desirability bias, which may have influenced responses to certain questions that relied on the respondents' memory. Additionally, the findings are based on self-reported data. However, several measures, such as quality control and observation checks, were incorporated into the questionnaire to ensure the accuracy of the collected data. Furthermore, the cross-sectional study design makes it impossible to establish a direct causal relationship between the predictor factors and the incidence of anemia.

## Conclusions

Anemia among children aged 6–59 months remains a severe public health problem in the Busoga Region of Uganda. Having a malaria infection and a child having a caretaker aged 45–59 years were positive predictors of anemia among children aged 6–59 months. Conversely, children aged 36–59 months were protected against anemia. Interventions such as the distribution of long-lasting insecticidal nets (LLINs), malaria vaccination, and chemoprophylaxis may help mitigate factors that lead to anemia. Integrated interventions that involve sensitizing child caretakers on optimal infant and young child feeding, socioeconomic empowerment, and water, sanitation, and hygiene (WASH) could also reduce the anemia burden in the region.

## Data Availability

The raw data supporting the conclusions of this article will be made available by the corresponding author without undue reservation.
